# Number of Facial Hair Corresponds to Frequency of Spontaneous Face‐Touch in Humans

**DOI:** 10.1002/adbi.202400243

**Published:** 2024-10-08

**Authors:** Martin Grunwald, Welda P. M. Pasatu, Jente Spille, Rene Haensel, Jens Stieler, Max Holzer, Mirjana Ziemer, Kevin H.G. Butz, Sven Martin, Stephanie Margarete Mueller, Markus Morawski

**Affiliations:** ^1^ Paul Flechsig Institute – Centre of Neuropathology and Brain Research Haptic Research Laboratory University of Leipzig 04103 Leipzig Germany; ^2^ Institute for Medical Informatics Statistics and Epidemiology (IMISE) University of Leipzig Härtelstr. 16‐18 D‐04107 Leipzig Germany; ^3^ Paul Flechsig Institute – Centre of Neuropathology and Brain Research University of Leipzig 04103 Leipzig Germany; ^4^ University of Leipzig Department of Dermatology Venereology and Allergology University Hospital Leipzig 04103 Leipzig Germany

**Keywords:** facial hair, human face, spontaneous facial self‐touches, vellus hair

## Abstract

People all over the world, independent of their culture or background, touch their faces up to 800 times per day. No other part of the body is touched as often as the face. Forehead, nose, and chin—the so‐called T‐zone of the face—are touched particularly frequently during spontaneous facial self‐touches (sFST). It is hypothesized that there is a relationship between the density of mechanoreceptors (inferred from facial hair distribution) and the frequency of spontaneous self‐touching. In order to indirectly measure the density of mechanoreceptors (cutaneous end organ complexes), the number of vellus and terminal hairs at 40 different measuring points on the face of 30 (15f/15m) healthy volunteers in study A is determined. In study B, the frequency of sFST at the same 40 measuring points in 66 (32f/34m) healthy persons is determined. Study A reveals that the number of facial hairs—in both sexes—is higher in the T‐zone than in other areas of the face. Study B reveals that the T‐zone is touched more frequently than other areas of the face. Skin areas of the face with a higher number of vellus hairs (and presumably higher innervation density) are touched particularly frequently during sFST.

## Graphical abstract

In this study, a bridge is built between the sensory innervation of the facial skin and spontaneous face touch. For the first time, a detailed analysis of the number of vellus and beard hairs on the human face is performed and extraordinary numbers are found. For example, there are ≈300 vellus hairs per 0.5 mm^2^ of each nostril (area: 8 × 6mm)!

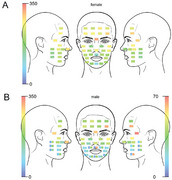

## 1 Introduction

**Figure 1:**
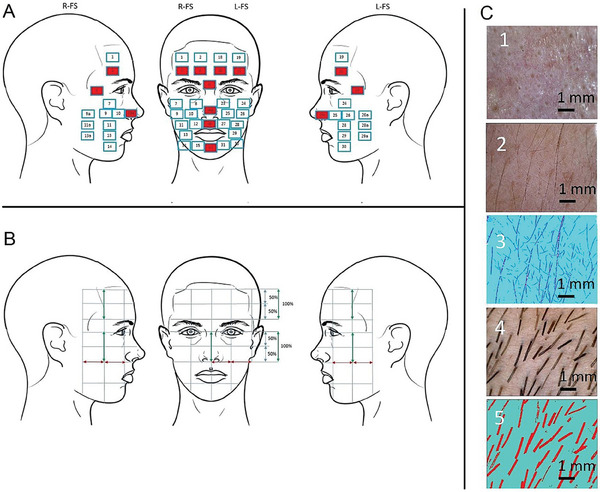
Schematic representation of the measuring points of the face and microscopic images. A,B) Schematic representation of the 40 measuring points for the microscopic imaging of the vellus hair. Measuring points marked in red (3, 4, 5, 6, 10a, 16a, 16, 17, 20, 21, 22, and 25a) represent the T‐zone. Laterally adjacent to the T‐zone are the right side of the face (R‐FS; measuring points 1, 2, 7, 8, 9a, 9, 10, 11a, 11, 12, 13a, 13, 14, and 15) and the left side of the face (L‐FS; measuring points 18, 19, 23, 24, 25, 26, 26a, 27, 28, 28a, 29, 29a, 30, and 31). C) Microscopic image of measuring point 5 (vellus hair) of a participant (male, 55 years). The size of the image area is 8.01 mm x 6.40 mm. 1) Before application of dye. 2) After application of dye. 3) Vellus hairs marked by the AI (Zeiss ZEN 3.4 Blue Edition). 4) Microscopic image of measuring point 15 (beard and vellus hair) of a male participant (34 years). The size of the image area is 8.01 mm x 6.40 mm. 5) Beard hairs marked by the AI (Zeiss ZEN 3.4 Blue Edition).

Current neurophysiological studies indicate that spontaneous facial self‐touches (sFST) serve regulatory processes of working memory and emotions.^[^ ^1^, ^2^, ^3^ ^]^ Analyses showed that humans touch their faces spontaneously and without directing attention to this behavior up to 400–800 times per day (16 waking hours).^[^ ^4^, ^5^ ^]^ Spontaneous touching of other body parts occurs less frequently than touching one´s own face. Moreover, sFST are performed equally often with the left and right hand and are directed equally often to the left (L‐FS) and right face side (R‐FS). However, the majority of sFST involve the facial T‐zone.^[^ ^4^, ^6^, ^7^, ^8^ ^]^ T‐zone refers to those areas of the face that include the midline (from the chin to the forehead) as well as the eyes and temple areas, thus involving the major facial body openings (see **Figure** **1**a). Therefore, touching one's own face can be associated with the transmission of pathogens to the mucous membranes of the mouth, nose, or eyes.^[^ ^9^, ^10^ ^]^

The psychological and physiological mechanisms underlying sFST are not yet fully understood. Furthermore, it remains unclear why most self‐touches involve the face rather than other body parts and why the majority of sFST involve the facial T‐zone. One potential cause may be the high innervation density of the facial skin. In the human facial skin, as in all areas of human hairy skin, there are various types of mechanoreceptors which include free nerve endings, Ruffini corpuscles, Meissner corpuscles, Merkel cell disks, and hair follicle receptors.^[^ ^11^, ^12^, ^13^ ^]^ The human face has the highest density of different mechanoreceptors, also called “cutaneous end organ complexes” or mechanosensory end‐organs ^[^ ^14^, ^15^, ^16^, ^17^, ^18^ ^]^ compared to other body areas with hairy skin. However, it is currently unknown whether the innervation density of the skin systematically varies between facial regions.

We suggest a relationship between the vellus hair density and presumably mechanoreceptor density and the frequency of sFST. The high receptor density of the facial skin might explain the dominance of sFST over other body areas. The high number of cutaneous end organ complexes in human facial skin may lead to a more pronounced biological processing of sensory stimulation of this area in comparison to other body parts.

Experimental assessments of tactile sensitivity, vibration perception, as well as pain and temperature perception indicate a higher sensitivity of the facial skin compared to the skin of other body parts.^[^ ^15^, ^18^ ^]^ Furthermore, sensitivity to mechanical, pain, and temperature stimuli is elevated in the oral region (corner of the mouth and lips) compared to the rest of the facial skin. In addition, the peripheral areas of the face and the forehead are less sensitive to tactile stimuli than the middle region of the face (mouth, lips, and nose). This effect may be due to a higher innervation density of the skin in the facial T‐zone than other facial areas. While the high neural density of the oral‐nasal region is well documented anatomically and histologically,^[^ ^19^, ^20^, ^21^ ^]^ a topographic analysis of the cutaneous end organ complexes of the entire face compared to the T‐zone is not yet available. Therefore, it is currently unknown if the T‐zone of the face has a higher mechanoreceptor density than other facial regions.

When testing the relationship between the cutaneous mechanosensory end organs density distribution in human facial skin and the frequency of sFST, methodological‐ethical challenges arise. In order to determine a representative topographical distribution of the mechanoreceptors of the facial skin in vivo, it would be necessary to take biopsy material from several points per face. As to the ethical‐human restrictions of biopsies, we chose an indirect and noninvasive method by determining the number of vellus and terminal hairs of the face in healthy persons using microscopic imaging. Because each hair is surrounded by multiple mechanoreceptors, the distribution density of mechanoreceptors in the facial skin can be inferred indirectly by counting the number of hairs. Small, short, and usually unpigmented vellus hairs as well as longer, thicker, and variable pigmented terminal hairs (such as scalp, eyelashes, and beard) can be found on human facial skin.^[^ ^22^ ^]^ Peters et al.^[^ ^23^ ^]^ also used a similar methodological procedure. An indirect measurement of Merkel receptor density was carried out by measuring the sweat pore density on the fingertips of humans.

Halata (1993) reported a receptor density per terminal hair of several dozen free nerve endings, up to 20 Merkel cells, and up to 50 lanceolate nerve terminals (sic.). For vellus hair, a receptor density of 1–5 free nerve endings is reported.^[^ ^24^ ^]^ To date, no topographical studies of the distribution density of hair on the human face exist. Previous authors have only investigated locally restricted segments of the face and body. For example, Blume et al. reported different average numbers of vellus hairs per cm^2^ in a selection of five body regions (forehead: 439 ± 23.7; cheek 416 ± 37.4; shoulder 68 ± 5.5, chest 57 ± 2.7; and back 85 ± 4.5).^[^ ^25^ ^]^ Mangelsdorf et al. investigated the number of hair follicles of different body regions (forehead, back, thorax, upper arm, forearm, thigh, calf) in male participants of different ethnic groups (Caucasian, African‐American, Asian). In all ethnicities, the authors found the highest number of hair follicles on the forehead compared to the other body regions.^[^ ^26^ ^]^

In the present study, the distribution of vellus hairs on the face will be investigated for both female and male participants based on 40 measuring points. Additionally, the number of terminal beard hairs will be assessed in male participants and added to the vellus hair count.

We tested the following hypotheses. Hypothesis 1: The T‐zone of the human face of both male and female participants contains a greater number of hairs than other areas of the face (right side of the face (R‐FS) and left side of the face (L‐FS)). Hypothesis 2: The majority of sFST are directed to facial areas, where most hairs are found. Hypothesis 1 was tested in study A, hypothesis 2 was tested in study B. Participants of study A did not participate in study B and vice versa. From a methodological perspective, because all healthy persons perform sFST and have vellus hair on the face, both hypotheses can be examined independently in different study groups.

## 2 Experimental Section

### 2.1 Study A: Facial Hair

#### 2.1.1 Participants

Thirty healthy, Caucasian volunteers took part in study A (15 females; age: *M* = 31.66, *SD* = 2.33; age range 22–50 years; 15 males; age: *M* = 31.66, *SD* = 2.72; age range 20–52 years). There was no significant age difference between participating males and females (Mann‐Whitney‐Test: *U* = 107.00; *Z* = −.229; *p* = .819). All participants were right‐handed according to a test of handedness.^[^ ^27^ ^]^ The inclusion criteria for male participants were defined as follows: no skin diseases, no shaving 4–5 days prior to the test, or a permanent 3‐day beard trimmed regularly. The inclusion criteria for female participants were defined as follows: no skin diseases, no facial makeup on the day of the study, and no shaving or plucking within the past 2 weeks. The study was approved by the Ethics Committee of the Medical Faculty of the University of Leipzig (462/21‐ek). Participants were faculty members and students. All participants signed informed consent. The participants received an expense allowance of 10€ h^−1^.

#### 2.1.2 Study Description

To obtain topographical comparability of the measuring points per participant, the face of each participant was marked with seven horizontal and seven vertical lines based on anatomical landmarks (**Figure** 1b). Based on these markings, a total of 40 measuring points were defined in the face (Figure 1a). The markings were drawn onto the participant's face by the experimenter using a cosmetic pen (Color Riche Le Khôl Superliner, L'Oréal Paris). To visualize the unpigmented vellus hairs each measuring point was dyed with an eyebrow tinting kit (SYOSS eyebrow kit, dark brown from Schwarzkopf & Henkel) (Figure 1c). Prior to the application of the dye, the face was covered with commercially available vaseline, to prevent staining of the skin. The dye was applied with a brush onto each measuring point. The exposure time of the dye was limited to 6 min, which resulted in stained vellus hair but unaffected skin. After this exposure time, the dye was washed off by the participants with warm water and soap. Afterward, the markings were drawn on the participant's face again, in order to identify the predefined measuring points more easily and with reliability during microscopic imaging. No measuring points were selected near the eyes in order to avoid injury during the application of the dye. In order to avoid skin reactions to the dye, all participants underwent a pretest 4 days prior to the examination. In this pretest, the dye was applied to the ventral forearm on an area of 1 cm^2^ for a period of 5 min. None of the participants showed skin reactions to the dye. During the examination, the participants were seated on a chair. The facial hairs of each measuring point were recorded with a digital microscopic camera (Micro Skale 2.0; dnt Germany) and subsequently marked and counted using an AI software (Zeiss, ZEN 3.4 Blue Edition, Module Intellesis). The Intellesis software module provides an automated machine‐learning and threshold‐based segmentation approach which allows large amounts of data to be evaluated and analyzed quickly and robustly.^[^ ^28^ ^]^

#### 2.1.3 Microscopy

The images of the measuring points were recorded with a digital microscope camera (Micro Skale 2.0; dnt Germany). The camera was connected to a laptop via USB and the software MicroCapture 2.5 (Informer Technologies, Inc.) was used to capture the images. The lighting of the measuring points was provided by an LED ring inside the microscope camera. The microscope camera was equipped with a custom‐made extension that framed an acrylic glass disk (inner diameter 25.16 mm, outer diameter 31.00 mm, thickness 2.00 mm). During image capture, the acrylic glass disk was pressed against the corresponding skin area to reduce any irregularities of the skin and push the hair flat against the skin. For each measuring point, a clean new acrylic glass disk was used. The image size per measuring point was (2560 × 2048 pixel; resolution 3.09 µm per pixel). The acrylic glass disks were cleaned in an ultrasonic bath using a 1% solution of Terg‐a‐zyme.

#### 2.1.4 Data Analysis Study A

##### Determination of Vellus and Beard Hairs

The number of vellus hairs in female and male participants was determined at the measuring points 1, 2, 3, 4, 5, 6, 7, 8, 9, 10, 10a, 16a,18, 19, 20, 21, 22, 23, 24, 25a, 25, 26 using Zen Zeiss 3.4, Blue Edition software (AI).^[^ ^29^ ^]^ In male participants, vellus and beard hair were counted separately for the measuring points 11, 12, 13, 14, 15, 16, 17, 27, 28, 29, 30, 31, 9a, 11a, 13a, 26a, 28a, and 29a using the AI software. The same software was used to count the vellus hairs of female participants on the measuring points 11, 12, 13, 14, 15, 16, 17, 27, 28, 29, 30, and 31. In contrast, measuring points 9a, 11a, 13a, 26a, 28a, and 29a of female participants could not be reliably determined by the AI. At these measuring points, the vellus hair length was significantly longer compared to other measuring points. The hair length resulted in intersections of multiple hairs that could not reliably be distinguished by the AI. Therefore, the mentioned measuring points were manually counted for all female participants by the raters M.G. and P.P. using the open‐source software ImageJ.^[^ ^30^ ^]^ The light background was substracted in each image using a rolling ball radius of 50 pixels prior to manual counting. The multi‐point function was used to indicate and count the hairs. Cronbach's α was 0.914.

Using 64 randomly selected images, the AI was trained with the characteristic values for vellus hairs (pixel >70, diameter <30 µm) and the characteristic values for beard hairs (pixel >300, diameter >60 µm). The detection of the vellus and beard hairs was carried out with separate AI modules. The vellus and beard hairs detected by the AI were color‐coded, see Figure 1c.

To determine the overall interrater reliability between raters M.G., P.P., and AI software, the vellus hairs of measuring points 4, 17, and 25a were counted manually by M.G. and P.P. for all participants. For measuring point 17, the number of vellus hairs and beard hairs was determined separately for male participants. Cronbach's α for M.G. and P.P. was 0.942. Cronbach's α for human versus machine was 0.646.

For the statistical comparison of the number of vellus hairs among T‐zone, right side, and left side of the face, the mean number of vellus hairs per facial area per person was determined. Non‐parametric Friedman tests were used for statistical comparisons of the number of vellus hairs between the three face areas across all participants and separately for male and female participants. Post‐hoc pairwise comparisons of the number of vellus hairs per facial area were performed separately for male and female participants, as well as across all participants using the non‐parametric Wilcoxon signed‐ranked test with Bonferroni‐corrected α level (0.05/9, *p*_crit_ = 0.005). Mann–Whitney *U*‐test was used to test for sex differences in the number of vellus hairs.

Additionally, explorative analyses were performed to investigate if differences between male and female facial hair distribution occured, when beard hair were added to the vellus hair count. Beard hair occured at measuring points 16 and 17 of the T‐zone, at measuring points 9a, 11a, 13a, 11, 12, 13, 14, and 15 of the right face side, and at measuring points 27, 28, 29, 30, 31, 26a, 28a, and 29a of the left face side.

### 2.2 Study B: Spontaneous Facial Self‐Touch (sFST)

#### 2.2.1 Participants

Ninety healthy, Caucasian volunteers (45 female; mean age: 26.52 years, SD: 4.79 years) participated in study B. All participants were right‐handed, according to a test of handedness.^[^ ^27^ ^]^ Based on self‐report, none of the participants reported any psychological, neurological, or psychiatric disorders. The participants were screened for the acute influence of 16 drugs with a urine test (Surestep Multi‐drug rapid test, Abbott). If the test was positive for any of the drugs, the screened person was excluded from further participating in the study. To prevent interfering cognitions about body movements and self‐touches, all participants were told that the study was focused on investigating working memory processes. After the experiment, all participants had the opportunity to ask questions. They received a reimbursement of 20 € h^−1^. All participants took part voluntarily and signed consent. The study was approved by the local ethics committee (426/20‐ek).

#### 2.2.2 Study Description

The frequency and location of sFST were assessed during a working memory experiment. Previous studies on sFST have shown that elaborate experimental conditions are required to obtain relevant numbers of sFST. Therefore, the present experiment consisted of four experimental blocks and each block consisted of a learning, a retention, and a reproduction phase. During each learning phase, the participants were presented with 20 words (semantic targets), which were displayed consecutively on a screen for one second. The words were different in each experimental block. Learning was followed by a 15‐min retention phase (RI), during which the participants had to remember the words. During the retention phase, half of the participants were in the distraction condition, the other half in the silence condition. The distraction condition consisted of randomized presentation of a fixation cross, auditory distractors, and semantic distractors. The silence condition consisted of the presentation of a blank screen during the entire retention phase. For each participant, the condition switched from block to block between distraction and silence, e.g., participants who were in the distraction condition during retention of block 1, were in the silence condition during retention of block 2. During the final phase of each block (reproduction), the participants had to write down the retained words from the learning phase of the respective experimental block.

Semantic targets and distractors were selected from the database Berlin Affective Word List Reloaded.^[^ ^31^ ^]^ Auditory distractors were selected from the database International Affective Digitized Sounds (IADS‐2).^[^ ^32^ ^]^

During the entire experiment, EEG, video, and electromyography (EMG) data were recorded, in order to track body movements and self‐touches. EEG electrodes were positioned according to the 10–20 system.^[^ ^33^ ^]^ EEG and EMG data were recorded with a sampling rate of 2000 Hz. The recording system (Brain Products, Germany) allowed parallel and synchronous recording of EMG, EEG, and video data. EEG results will be presented elsewhere. EMG electrodes were positioned on the arms (*m. extensor carpi ulnaris*), legs (*m. tibialis anterior*), the right lower back (*m. latissimimus dorsi*), and the right neck (*m. trapezius*) of the participants. For facial self‐touches, the parameters of interest were frequency and touched face area.

#### 2.2.3 Data Analysis Study B

A subgroup of *n* = 66 participants (34 men/32 women; age: M = 25.73, SD = 3.34) performed sFST during the RI. There was no significant age difference between male and female participants (Mann–Whitney *U*‐tests; *U* = 228.00, *Z* = −0.349, *p* = 0.727). Video data from these 66 participants were used to register facial touches according to the 40 measuring points in Figure 1a. Two independent raters (M.A. and C.T.S.) determined the number of sFST per participant according to the previously defined 40 measuring points (see Figure 1a). Cronbach's α was 0.79. The mean number and standard deviation (SD) of sFST per measuring point are shown for the whole group, as well as for the male and female subgroups separately in Table S1 (Supporting Information).

For the statistical comparison between sFST frequency of T‐zone, R‐FS, and L‐FS, the mean number of sFST per facial area and participant was determined. Non‐parametric Friedman tests were used for statistical comparisons of the number of sFST between the three face areas for male and female participants as well as across all participants. Post hoc pairwise comparisons of the number of sFST per facial area were performed separately for males and females as well as across all participants using the non‐parametric Wilcoxon signed‐ranked test with Bonferroni‐corrected α level (0.05/9, *p*_crit_ = 0.005). Mann–Whitney *U*‐test was used to test for sex differences in the number of sFST. The Cohen's D effect size was reported for each test. SPSS Software 27^[^ ^34^ ^]^ was used for all statistical analyses of studies A and B.

## 3 Results

### 3.1 Study A: More Hairs in the T‐Zone Than on the Left and Right Sides of the Face

**Figure 2:**
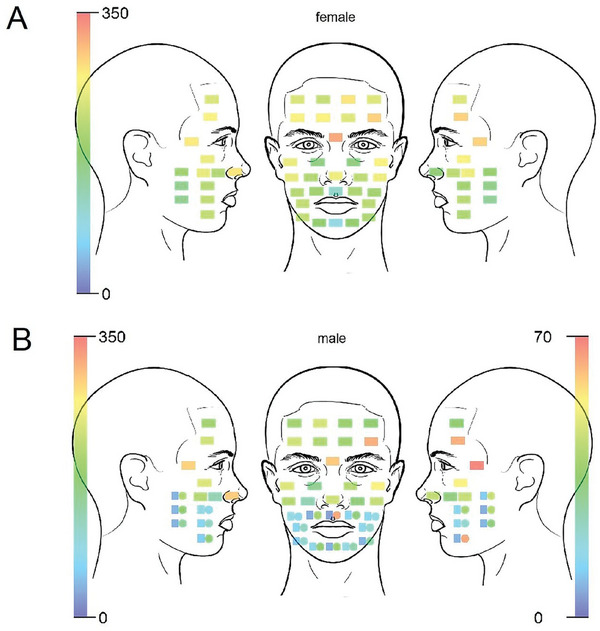
Distribution of vellus hair in female and male participants. A) Mean number of vellus hair (scale on left side) and beard hair (scale on right side) in male participants per measuring point. B) Squares display the amount of vellus hair and circles display the amount of beard hair.

Descriptive results of the numbers of vellus hairs and terminal beard hairs per face area are reported in **Table** **1**. The number of vellus hairs per measuring point for the entire group as well as for the female and male participants are reported in Table S2 (Supporting Information). Measuring points 6, 21, and 22 had the highest mean number of vellus hair in both male (*M *= 319.40, SD = 21.88) and female participants (*M* = 302.44, SD = 16.13; see **Figure** **2**).

**Table 1:** Descriptive statistics of vellus hairs and beard hairs.

	All participants (vellus)			Female (vellus)			Male (vellus)			Male (beard hair)		
Face area	MD	Min	Max	MD	Min	Max	MD	Min	Max	MD	Min	Max
T‐zone	235.83	111.58	350.08	247.41	111.58	339.58	228.66	155.50	350.08	55.00	14.50	80.50
R‐FS	152.01	68.14	279.25	218.17	123.71	279.25	109.78	68.14	227.00	28.75	13.38	40.00
L‐FS	158.01	66.86	275.25	231.85	100.54	275.25	121.85	66.86	211.07	33.00	12.00	41.13

Median (MD), minimum (min), and maximum (max) of the frequency of spontaneous facial self‐touches within the T‐zone, the right side of the face (R‐FS), and the left side of the face (L‐FS) for all participants as well as for the male and female subgroups.

Comparisons between male and female participants revealed no statistically significant difference in the number of vellus hairs in the T‐zone (Mann–Whitney *U*‐test: *Z* = −0.892, *p* = 0.373, Cohen‘s *d* = 0.33). However, female participants had significantly more vellus hairs than male participants on the right (*Z* = −3.920, *p* = 0.001, Cohen‘s *d* = 2.049) and left side of the face (*Z* = −3.629, *p* = 0.001, Cohen‘s *d* = 1.769; see also Table S4. When the beard hairs were added to the vellus hair count, the same differences remained statistically significant as when only vellus hair numbers were compared: female participants had significantly more hair on the right and left side of the face than male participants but there was no sex difference in the number of hair in the T‐zone (Table S4, Supporting Information). The distribution of vellus hair in female participants is shown in Figure 2a. The distribution of vellus and beard hairs in male participants is shown in Figure 2b.

Concerning hypothesis 1, analyses revealed a significant difference in the number of vellus hairs between the three facial areas for the whole group (Chi^2^ = 31.267, *p* = 0.001, *r* = 1.0209) as well as for the male (Chi^2^ = 22.800, *p* = 0.001, r = 1.2329) and female subgroups (Chi^2^ = 10.133, *p* = 0.006, *r* = 0.8219). The post‐hoc pairwise Wilcoxon tests showed significantly higher numbers of vellus hairs in the T‐zone than on the right and left side of the face for the whole group as well as for male and female participants (see Table S5, Supporting Information). Furthermore, the number of vellus hairs did not differ between the right and left side of the face, neither in male nor female participants (see Table S5, Supporting Information). Therefore, the mean number of vellus hairs in the facial T‐zone is higher than on the left and right sides of the face in both male and female participants. These results remained unchanged when both vellus and beard hair were included in the analyses (see Table S5, Supporting Information).

### 3.2 Study B: T‐Zone is Touched More Frequently Than Right or Left Side of Face

**Figure 3:**
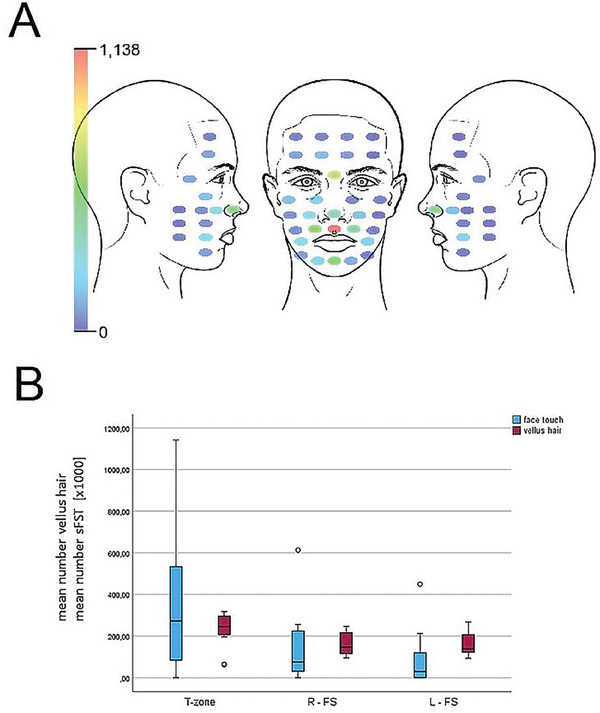
Number of spontaneous facial self‐touches and vellus hair per face area. A) Heat map of mean number of spontaneous facial self‐touches per measuring point for all participants. B) Boxplot of number of vellus hair and mean number of facial self‐touch per face area. Face areas: T‐zone, right side of the face (R‐FS), and left side of the face (L‐FS). Mean numbers of spontaneous facial self‐touch (sFST) were multiplied by 1000.

Descriptive statistics of sFST per face area are reported in **Table** **2**. Statistical analyses revealed significant differences in the frequency of facial self‐touch between the face areas for the whole group (Chi^2^ = 49.441, *p* = 0.001, Kendall‐W = 0.375) as well as for the male (Chi^2^ = 26.804, *p* = 0.001, Kendall‐W = 0.394) and for the female subgroup (Chi^2^ = 22.709, *p* = 0.001, Kendall‐*W* = 0.355). The frequency of T‐zone touch was significantly higher than the touch frequency of the right or left face side (see Table 2 and **Figure** **3**a). Furthermore, the right side of the face was touched significantly more often than the left side (see Table 2 and Table S3, Supporting Information).

**Table 2:** Descriptive statistics of spontaneous facial self‐touches.

	All participants			Male (*n* = 34)			Female (*n* = 32)		
Face area	MD	Min	Max	MD	Min	Max	MD	Min	Max
T‐zone	2.00	0.00	21.17	2.00	0.00	19.08	2.08	0.00	21.17
R‐FS	1.00	0.00	32.14	0.50	0.00	32.14	1.00	0.00	6.07
L‐FS	0.07	0.00	20.07	0.03	0.00	20.07	1.00	0.00	5.00

Median (MD), minimum (min), and maximum (max) of the frequency of spontaneous facial self‐touches within the T‐zone, the right side of the face (R‐FS), and the left side of the face (L‐FS) for all participants as well as for the male and female subgroups.

Male and female participants did not differ in their sFST frequency in the T‐zone (*Z* = −0.679, *p* = 0.497, Cohen‘s d = 0.166), right face side (*Z* = −0.305, *p* = 0.760, Cohen‘s *d* = 0.071), or left face side (*Z* = −0.542, *p* = 0.588, Cohen‘s *d* = 0.125).

In combination, the results of the two studies indicate a similar trend in the number of facial hairs and the number of sFST per facial area (Figure 3b).

## 4 Discussion

The aim of the present experiments was to investigate a potential association between the location of sFST and innervation density of the facial skin. Study A examined the hair density in the human face on 40 preselected measuring points as an approximation of in vivo mechanoreceptors distribution. Previous studies indicated a higher innervation density of the face, and especially of the oral region, compared to other body parts.^[^ ^15^, ^16^, ^17^, ^18^ ^]^ However, no systematic analyses of the entire face existed. Therefore, we aimed to investigate the number of vellus hairs of the facial skin, in both male and female participants. We assumed that the number of hairs should differ between the T‐zone and the right and left sides of the face (Hypothesis 1). The total number of measuring points was 40, with 12 in the T‐zone, and 13 per left and right side of the face. The analyses confirmed Hypothesis 1 and showed that both sexes had a higher number of vellus hairs in the T‐zone compared to the right or left side of the face. Hypothesis 1 was also confirmed when beard hair were added to the vellus hair count; accordingly, the total number of facial hair (beard plus vellus hairs) was higher in the T‐zone than on the left and right side of the face indicating an increased innervation density in this particular facial skin area. Higher innervation density is a prerequisite for more pronounced biological processing of sensory stimulation, which has previously been hinted at by smaller perceptual thresholds of the facial midline compared to other face areas^[^ ^35^ ^]^ The T‐zone includes vulnerable regions (eyes, mouth, and nose) that necessitate protection from external factors. High sensory innervation of these potential gateways ensures increased mechanosensory sensitivity and safeguarding. Similarly, checking for foreign materials or foreign organisms in the mouth, nose, or eyes region might at least partially account for the high rate of sFST directed to the T‐zone.

The median number of vellus hairs in the T‐zone did not differ between the two sexes, yet significant differences between the sexes were observed when comparing the number of vellus hairs on the right side (R‐FS) and the left side of the face (L‐FS) (Table 1). Female participants have significantly more vellus hairs on the right and left side of the face than the male participants do, which may be explained by the regular shaving rituals of male participants. Male participants were instructed to abstain from shaving for at least four days prior to the experiment, however, vellus hair grow much slower than beard hair and may have been too short to register.^[^ ^25^ ^]^ However, when beard hair were added to the vellus hair count, male participants still showed less total facial hair on the left and right face side than female participants (see Table S4, Supporting Information).

The higher amount of vellus hairs in the T‐zone area of both sexes may be associated with the fact that two orifices (mouth, nose) as well as the eyes are located in this facial area. The increased number of vellus hairs may ensure an increased “mechanosensitive protection” of these vulnerable mucous membranes. In contrast, the sex difference in the number of vellus hairs on the R‐FS and L‐FS cannot be explained, yet. According to Beek male persons are generally more hairy after puberty than females.^[^ ^36^ ^]^ According to this finding, it would be expected that male persons would have more facial hairs. However, our results showed that male participants exhibit less facial hairs (vellus plus beard hairs) than female participants on the right and left side of the face (see Table 1). It is unlikely that age can explain the difference in the number of facial hair between sexes, because there was no age difference between the groups. We found (see Table S2, Supporting Information) a small number of vellus hairs in the area of the mouth—especially at measuring points 16 and 17. This result is somewhat contradictory to the studies that show that the mouth region is particularly strongly innervated.^[^ ^20^ ^]^ We suspect that the density of mechanosensory units in the mouth region is very high, especially on or just around the lips. However, the position of our measuring points did not include the lip vermilion and its border.^[^ ^37^ ^]^

The aim of study B (Hypothesis 2) was to examine whether the majority of sFST are directed to facial areas, where most hairs are found. In support of the hypothesis, the results showed significantly higher sFST frequency in the T‐zone than on the right or left side of the face. The sFST frequency did not differ between the right and left sides of the face. Furthermore, there were no differences in touch frequency between male and female participants for the T‐zone, R‐FS, and L‐FS. These findings are consistent with previous studies, demonstrating that sFST are performed more frequently in the T‐zone compared to other areas of the face.^[^ ^4^, ^5^, ^6^, ^7^, ^8^ ^]^ Both in everyday situations (e.g., driving a car) and during experimental settings, the T‐zone is the area of the human face that is most frequently touched during sFST. Therefore, phenomenologically, a body part with very high innervation density (finger) is used to touch another densely innervated skin region (facial T‐zone). It can be assumed that the sensory and neural response of touch between two densely innervated body parts is quite large, even if the touch is performed with low force and short duration. In light of current EEG findings that showed that spontaneous facial self‐touches serve regulatory processes of working memory and emotions.^[^ ^1^, ^2^, ^3^ ^]^ Further investigations of facial sensory innervation and their association with cortical processing should be conducted.

### 4.1 Limitations

It is not yet clear when and why the different distributions of vellus hair develop in the human face. It is also unclear whether frequent mechanical stress of the skin in the T‐zone leads to increased growth of vellus hairs in this area or if the characteristic distribution of the vellus hairs of adults—in both sexes—may already exist in children or even in the fetus. Despite the relatively large number of measuring points, only a fraction of the facial skin area was covered by the microscopy method we used. Moreover, the microscopy tool could not reach all areas of the nose. In order to further improve the representativeness of the results, optical measurement methods should be developed and used in subsequent studies that can capture a larger number of measurement points per face.

The frequency of spontaneous facial touching and the amount of vellus hair within the T‐zone is not equally distributed. The T‐zone includes 11 measuring points (3, 4, 5, 6, 10, 16a, 16, 17, 21, 22, and 25a). The measuring points of the mouth region (16, 17) are most frequently touched in the context of sFST, but have relatively few vellus hairs. On the other hand, measuring points on the forehead (4, 20), show a high number of vellus hairs and are touched relatively little during sFST (see Tables S1 and S2, Supporting Information). The vellus hair count does not include an estimate of mechanosensory end‐organs of hairless areas (e.g., vermilion and cornea). Lips and eyes are located within the T‐zone and have been shown to be densely innervated.^[^ ^37^ ^]^ However, our current measurement method was not suitable to include these regions. Therefore, we suspect that the unequal distribution of sFST and estimated mechanoreceptor density may be at least partially due to the chosen method

Even if it is ethically and methodologically very demanding, future research should strive to analyze the skin's cutaneous end organ complexes density by using biopsy material from several facial areas. The work of Nolano et al. is an important step in this direction^[^ ^12^ ^]^ These analyses can also elucidate which mechanosensory units are distributed in the facial skin in addition to the mechanosensitive innervation of facial hairs. Investigating the cutaneous end‐organ complexes distribution of the face is the only way to better understand the facial skin as a complex sensory organ.

## 5 Conclusion

The facial T‐zone has a higher number of vellus hair than the left and right sides of the face. Those skin areas of the face that have probably a high innervation density of cutaneous end organ complexes are touched particularly frequently during sFST.

## Conflict of Interest

The authors declare no conflict of interest.

## Author Contributions

M.G., S.M.M., and M.M. designed research; W.P.M.P., J.P., R.H., K.H.G.B., and S.M. performed research; M.G., W.P.M.P., S.M.M., and M.Z. analyzed data; M.G., J.S., M.H., M.Z., S.M.M., M.M., and K.H.G.B wrote the paper.

## Supporting information



Supporting Information

## Data Availability

The data that support the findings of this study are available in the supplementary material of this article.
