# Supplementary material for: Number of Facial Hair Corresponds to Frequency of Spontaneous Face‐Touch in Humans
Source: Adv Biol (Weinh). 2024 Oct 8;9(2):2400243. doi: 10.1002/adbi.202400243 (PMC11830429; doi:10.1002/adbi.202400243)
Supplement: Supplementary file 1 — Supporting Information [file ADBI-9-2400243-s001.docx]

**Supporting Information**

Table S1

Facial self-touch frequency

Mean frequencies and standard deviation (SD) of the facial self-touch frequency at each measuring point per group, male, female.

| facial measuring point | group | | male | | female | |
| --- | --- | --- | --- | --- | --- | --- |
|  | mean | SD | mean | SD | mean | SD |
| 1 | .03 | .25 | .06 | .34 | .00 | .00 |
| 2 | .03 | .17 | .03 | .17 | .03 | .18 |
| 3 | .09 | .29 | .09 | .29 | .09 | .30 |
| 4 | .15 | .50 | .18 | .52 | .13 | .49 |
| 5 | .11 | .31 | .06 | .24 | .16 | .37 |
| 6 | .73 | 1.47 | .65 | 1.72 | .81 | 1.18 |
| 7 | .17 | .41 | .06 | .24 | .28 | .52 |
| 8 | .21 | .62 | .21 | .59 | .22 | .66 |
| 9 | .06 | .24 | .09 | .29 | .03 | .18 |
| 10 | .26 | .56 | .29 | .68 | .22 | .42 |
| 11 | .06 | .30 | .09 | .38 | .03 | .18 |
| 12 | .62 | 2.32 | .88 | 3.17 | .34 | .65 |
| 13 | .26 | 1.29 | .44 | 1.78 | .06 | .25 |
| 14 | .09 | .34 | .12 | .41 | .06 | .25 |
| 15 | .23 | .65 | .29 | .80 | .16 | .45 |
| 16 | 1.14 | 1.75 | .97 | 1.57 | 1.31 | 1.94 |
| 17 | .55 | 1.13 | .32 | .81 | .78 | 1.36 |
| 18 | .05 | .21 | .00 | .00 | .09 | .30 |
| 19 | .00 | .00 | .00 | .00 | .00 | .00 |
| 20 | .08 | .27 | .03 | .17 | .13 | .34 |
| 21 | .00 | .00 | .00 | .00 | .00 | .00 |
| 22 | .06 | .24 | .09 | .29 | .03 | .18 |
| 23 | .03 | .17 | .06 | .24 | .00 | .00 |
| 24 | .03 | .17 | .03 | .17 | .03 | .18 |
| 25 | .17 | .41 | .15 | .36 | .19 | .47 |
| 26 | .00 | .00 | .00 | .00 | .00 | .00 |
| 27 | .45 | 1.91 | .62 | 2.57 | .28 | .73 |
| 28 | .03 | .17 | .06 | .24 | .00 | .00 |
| 29 | .21 | .60 | .21 | .59 | .22 | .61 |
| 30 | .02 | .12 | .00 | .00 | .03 | .18 |
| 31 | .12 | .33 | .15 | .36 | .09 | .30 |
| 91 | .00 | .00 | .00 | .00 | .00 | .00 |
| 10a | .52 | 1.14 | .53 | .96 | .50 | 1.32 |
| 11a | .00 | .00 | .00 | .00 | .00 | .00 |
| 13a | .03 | .17 | .03 | .17 | .03 | .18 |
| 16a | .39 | .80 | .38 | .78 | .41 | .84 |
| 25a | .50 | .81 | .50 | .90 | .50 | .72 |
| 26a | .00 | .00 | .00 | .00 | .00 | .00 |
| 28a | .00 | .00 | .00 | .00 | .00 | .00 |
| 29a | .02 | .12 | .03 | .17 | .00 | .00 |

Table S2

Number of vellus hairs

Number of vellus hairs for the total group (mean, SD) as well as for male and female participants per measuring point

| facial measuring  point | group | | male | | female | |
| --- | --- | --- | --- | --- | --- | --- |
|  | mean | SD | mean | SD | mean | SD |
| 1 | 216.00 | 131.32 | 193.07 | 165.64 | 238.93 | 84.59 |
| 2 | 229.13 | 99.20 | 233.93 | 93.30 | 224.33 | 107.85 |
| 3 | 249.17 | 104.12 | 230.87 | 104.12 | 267.47 | 104.39 |
| 4 | 237.43 | 106.97 | 204.67 | 77.74 | 270.20 | 123.93 |
| 5 | 284.70 | 82.81 | 293.40 | 72.96 | 276.00 | 93.38 |
| 6 | 310.20 | 86.16 | 299.33 | 86.01 | 321.07 | 87.90 |
| 7 | 246.47 | 71.16 | 241.40 | 74.47 | 251.53 | 69.92 |
| 8 | 155.17 | 83.62 | 149.53 | 62.86 | 160.80 | 102.30 |
| 9 | 230.03 | 78.39 | 210.47 | 79.47 | 249.60 | 74.79 |
| 10 | 167.13 | 77.90 | 126.87 | 59.00 | 207.40 | 74.95 |
| 11 | 148.41 | 94.35 | 85.60 | 77.63 | 211.21 | 62.95 |
| 12 | 113.03 | 98.39 | 35.13 | 35.75 | 190.93 | 75.98 |
| 13 | 143.23 | 102.60 | 66.80 | 60.41 | 219.67 | 75.10 |
| 14 | 139.23 | 101.98 | 56.07 | 54.02 | 222.40 | 61.69 |
| 15 | 125.47 | 84.81 | 75.27 | 79.55 | 175.67 | 56.30 |
| 16 | 66.23 | 62.96 | 25.67 | 46.65 | 106.80 | 50.08 |
| 17 | 61.23 | 57.10 | 35.87 | 33.14 | 86.60 | 65.40 |
| 18 | 231.93 | 121.34 | 184.40 | 67.70 | 279.47 | 145.17 |
| 19 | 207.47 | 90.12 | 172.93 | 64.64 | 242.00 | 100.45 |
| 20 | 217.10 | 77.27 | 191.47 | 69.32 | 242.73 | 78.46 |
| 21 | 304.63 | 127.86 | 316.13 | 146.47 | 293.13 | 110.11 |
| 22 | 317.93 | 98.11 | 342.73 | 112.77 | 293.13 | 76.82 |
| 23 | 187.57 | 137.31 | 210.87 | 172.87 | 164.27 | 89.50 |
| 24 | 267.20 | 88.61 | 268.47 | 89.53 | 265.93 | 90.80 |
| 25 | 190.27 | 90.73 | 164.67 | 72.71 | 215.87 | 101.79 |
| 26 | 238.67 | 82.23 | 223.40 | 94.67 | 253.93 | 67.42 |
| 27 | 135.50 | 103.87 | 67.73 | 64.58 | 203.27 | 91.30 |
| 28 | 130.83 | 91.85 | 68.60 | 62.43 | 193.07 | 72.66 |
| 29 | 143.30 | 93.36 | 73.07 | 56.30 | 213.53 | 65.69 |
| 30 | 121.80 | 103.06 | 40.47 | 30.97 | 203.13 | 82.87 |
| 31 | 131.83 | 82.48 | 86.07 | 68.89 | 177.60 | 69.69 |
| 91 | 115.25 | 91.06 | 39.27 | 33.79 | 191.23 | 60.53 |
| 10a | 288.70 | 203.54 | 297.20 | 205.69 | 280.20 | 208.22 |
| 11a | 97.62 | 71.31 | 41.27 | 37.86 | 153.97 | 47.93 |
| 13a | 94.37 | 66.70 | 37.93 | 30.93 | 150.80 | 37.88 |
| 16a | 242.30 | 95.73 | 230.00 | 78.40 | 254.60 | 111.87 |
| 25a | 195.77 | 100.96 | 220.20 | 100.57 | 171.33 | 98.60 |
| 26a | 118.87 | 82.20 | 55.73 | 57.12 | 182.00 | 46.85 |
| 28a | 96.92 | 75.50 | 34.93 | 37.84 | 158.90 | 46.29 |
| 29a | 93.18 | 63.52 | 40.73 | 35.96 | 145.63 | 34.21 |

Table S3

Statistical comparisons of the spontaneous facial self-touch frequency in different face areas.

|  | all participants | | | male (*n* = 34) | | | female (*n* = 32) | | |
| --- | --- | --- | --- | --- | --- | --- | --- | --- | --- |
| Face area | *Z* | *p* | d_Cohen_ | *Z* | *p* | d_Cohen_ | *Z* | *p* | d_Cohen_ |
| T-zone vs. R-FS | -4.318 | .001 | 1.255 | -2.516 | .012 | 0.957 | -3.578 | .001 | 1.633 |
| T-zone vs. L-FS | -5.790 | .001 | 2.032 | -3.976 | .001 | 1.864 | -4.265 | .001 | 2.295 |
| L-FS vs. R-FS | -2.632 | .008 | 0.685 | -2.114 | .035 | 0.778 | -1.540 | .124 | 0.566 |

Table S4

Statistical comparisons of facial hair distribution between female and male participants.

|  | female [vellus]  vs.  male [vellus] | | | female [vellus]  vs.  male [vellus+beard] | | |
| --- | --- | --- | --- | --- | --- | --- |
| Face area | *Z* | *p* | d_Cohen_ | *Z* | *p* | d_Cohen_ |
| T-zone | -0.892 | .373 | 0.33 | -0.394 | .713 | 0.14 |
| R-FS | -3.920 | .001 | 2.04 | -3.380 | .001 | 1.56 |
| L-FS | -3.629 | .001 | 1.76 | -3.215 | .001 | 1.45 |

Table S5

Statistical comparison of the number of facial hairs in different face areas.

|  | all participants | | | male (*n* = 15) | | | female (*n* = 15) | | |
| --- | --- | --- | --- | --- | --- | --- | --- | --- | --- |
| 1. Vellus hair only | | | | | | | | | |
| Face area | *Z* | *p* | d_Cohen_ | *Z* | *p* | d_Cohen_ | *Z* | *p* | d_Cohen_ |
| T-zone vs. R-FS | -4.618 | .000 | 3.316 | -3.408 | .001 | 1.59 | -2.953 | .003 | 1.280 |
| T-zone vs. L FS | -4.576 | .000 | 3.041 | -3.408 | .001 | 1.59 | -2.840 | .005 | 1.213 |
| L-FS vs. R-FS | -1.409 | .159 | 0.532 | -1.193 | .233 | 0.44 | -0.966 | .334 | 0.358 |
| 1. Vellus plus beard hair | | | | | | | | | |
|  | Z | p | dCohen | Z | p | dCohen | - | - | - |
| T-zone vs. R-FS | -4.618 | .000 | 3.316 | -3.408 | .001 | 1.59 | - | - | - |
| T-zone vs. L FS | -4.576 | .000 | 3.041 | -3.408 | .001 | 1.59 | - | - | - |
| L-FS vs. R-FS | -1.471 | .141 | 0.558 | -1.136 | .256 | 0.42 | - | - | - |

R-FS = right face side, L-FS = left face side. 1) z = Wilcoxon-test. Effekt sizes (Cohen’s d) are reported for each test; 2) z = Mann-Whitney-U-test. Left collumn: vellus hair distribution per face area; right collumn: vellus plus beard hair per face area. Effekt sizes (d_Cohen_) are reported for each test. 3) z = Wilcoxon-test, effekt sizes (dCohen) are reported for each test. a) Statistical comparison of the number of vellus hairs in the T-zone, right (R-FS) and left side of the face (L-FS) for all participants and for the male and female subgroups separately (Wilcoxon test). b) Statistical comparison of the number of all facial hair (vellus plus beard hairs) in the T-zone, right (R-FS) and left side of the face (L-FS) for all participants and for the male separately (Wilcoxon test).
